# Supplementary material for: The interactome of intact mitochondria by cross-linking mass spectrometry provides evidence for coexisting respiratory supercomplexes
Source: Mol Cell Proteomics. 2017 Dec 8;17(2):216–32. doi: 10.1074/mcp.RA117.000470 (PMC5795388; doi:10.1074/mcp.RA117.000470)
Supplement: Supplemental Data [file supp_RA117.000470_133922_0_supp_23978_fzffwf.zip › spectra_annotation/mito_DR_spectra_annotation/371-1-12-1-1-1.pdf]

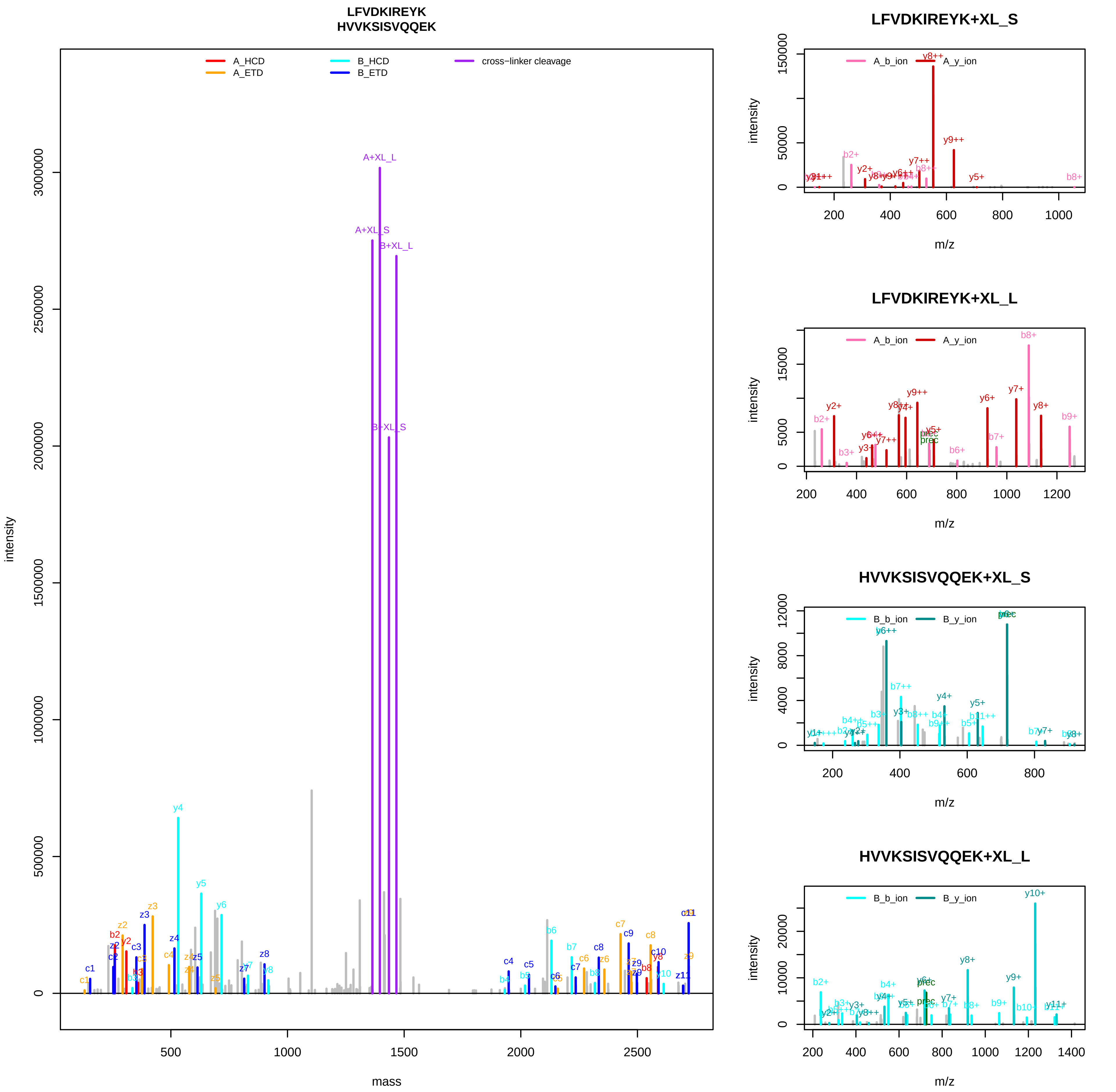

LFVDKIREYK
HVVKSISVQQEK
A_HCD
A_ETD
B_HCD
B_ETD
cross−linker cleavage
A+XL_L
A+XL_S
B+XL_L
B+XL_S
y4
y5
y6
intensity
mass
0
500000
1000000
1500000
2000000
2500000
3000000
500
1000
1500
2000
2500
LFVDKIREYK+XL_S
A_b_ion
A_y_ion
y8++
y9++
y7++
y5+
b2+
b8+
intensity
m/z
0
50000
150000
200
400
600
800
1000
LFVDKIREYK+XL_L
A_b_ion
A_y_ion
b8+
y9++
y7+
y6+
y8+
b9+
y2+
b2+
0
5000
15000
200
400
600
800
1000
1200
HVVKSISVQQEK+XL_S
B_b_ion
B_y_ion
y6++
b7++
y4+
y5+
0
4000
8000
12000
200
400
600
800
HVVKSISVQQEK+XL_L
B_b_ion
B_y_ion
y10+
y8+
y9+
b2+
b4+
0
10000
20000
200
400
600
800
1000
1200
1400
